# Enterovirus genomic load and disease severity among children hospitalised with hand, foot and mouth disease

**DOI:** 10.1016/j.ebiom.2020.103078

**Published:** 2020-11-06

**Authors:** Chunlan Song, Yu Li, Yonghong Zhou, Lu Liang, Lance Turtle, Fang Wang, Peng Wu, Qi Qiu, Jianli Yang, Kai Wang, Peng Cui, Yibing Cheng, Tianchen Zhang, Chun Guo, Mengyao Zeng, Lu Long, Malik Peiris, Chongchen Zhou, Benjamin J Cowling, Hongjie Yu

**Affiliations:** a Children's Hospital Affiliated to Zhengzhou University, Henan Children's Hospital, Zhengzhou, China; b WHO Collaborating Centre for Infectious Disease Epidemiology and Control, School of Public Health, Li Ka Shing Faculty of Medicine, The University of Hong Kong, Hong Kong Special Administrative Region, China; c Division of Infectious Disease, Key Laboratory of Surveillance and Early-warning on Infectious Disease, Chinese Center for Disease Control and Prevention, Beijing, China; d School of Public Health, Fudan University, Key Laboratory of Public Health Safety, Ministry of Education, Shanghai, China; e West China School of Public Health and West China Fourth Hospital, Sichuan University, Chengdu, China; f NIHR Health Protection Research Unit for Emerging and Zoonotic Infections, University of Liverpool, Liverpool, United Kingdom; g Tropical & Infectious Disease Unit, Royal Liverpool University Hospital (member of Liverpool Health Partners), Liverpool, United Kingdom; h School of Public Health, Huazhong University of Science and Technology, Wuhan, China; i NHC key Lab of Reproduction Regulation (Shanghai Institute of Planned Parenthood Research), Medical School, Fudan University, Shanghai, China

**Keywords:** HFMD, Viral load, Viral genomic load, Enterovirus, Clinical severity

## Abstract

**Background:**

Examining associations between viral genomic loads of enteroviruses and clinical severity is important for promoting and improving development of antiviral drugs related to hand, foot and mouth disease (HFMD).

**Methods:**

Throat swabs were collected from HFMD cases at acute phase of illness using a standardized technique in a prospective study. The viral genomic load was categorized into low, medium, and high groups using parameters of real-time reverse transcription-polymerase chain reaction. The clinical severities were assessed with four indicators, respectively.

**Findings:**

We analysed 1109 HFMD cases, including 538 children with CV-A6, 231 with CV-A16, 156 with EV-A71, 78 with CV-A10, 59 with CV-A4, and 47 with CV-A2. EV-A71 genomic load categories were associated with risks of diagnoses of CNS complications (*p* = 0.016), requiring systemic corticosteroids or IVIG (*p* = 0.011), intensive care unit admission (*p* = 0.002) and length of hospital stay over 5 days (*p* = 0.048). In the multivariate analyses, point estimates of adjusted odds ratio (OR) tended to increase with viral genomic loads for all four severe outcomes and ORs of highest viral genomic load were all significantly larger than one for EV-A71.

**Interpretation:**

HFMD clinical severities positively associate with viral genomic loads of EV-A71 in throat swabs. Specific antiviral drugs should be developed to reduce enterovirus load and to alleviate the clinical severities for HFMD cases.

**Funding:**

National Science Fund for Distinguished Young Scholars

**Research in context**

**Evidence before this study**

We searched PubMed for the articles on association between viral load and clinical severity of hand, foot and mouth disease (HFMD), using the search terms "hand, foot, and mouth disease", "EV71", "enterovirus 71″, "enterovirus A71", "coxsackie virus A", "viral load", "virus load" and "genomic load" without language restrictions. The searched articles were required to be published from January 1, 1994 to August 30, 2020. There were animal model studies suggesting the decrease in viral load of EV-A71 could lead to the decline in case fatality risk, while there was an English study reporting severe EV-A71 infections that were related to prolonged EV-A71 viremia in patients. A single small Chinese study compared EV-A71 viral load in 20 HFMD patients, in which no statistically significant difference was detected between mild and severe cases.

**Added value of this study**

This study examined the association between enterovirus viral load in throat swabs and clinical severity by enterovirus serotype among inpatient HFMD cases. We found the evidence that the risks of developing severe HFMD was positively associated with the viral loads of enterovirus for patients with EV-A71.

**Implications of all the available evidence**

Viral loads of EV-A71 were closely related to disease progression and deterioration in HFMD. Specific antiviral drugs should be developed to reduce EV-A71 viral load. The early use of effective antiviral drugs might improve the clinical outcome of HFMD patients with EV-A71.

Alt-text: Unlabelled box

## 1 Introduction

Hand, foot and mouth disease (HFMD) is an infectious disease mostly affecting infants and children under 5 years [1]. HFMD is generally a benign self-limited disease, with most cases presenting merely with exanthema, fever and other mild clinical manifestations, and recovering within several days. However, in a minority of HFMD cases, central nervous system (CNS) complications develop, which may progress to cardiopulmonary collapse and death. Surviving cases can suffer long term sequelae [1]. HFMD is caused by human enteroviruses, which are positive-sense single stranded RNA viruses of about 27–30 nm in size [2]. The HFMD etiological agents primarily belong to species of Enterovirus (EV) A, including commonly detected serotypes of EV-A71, coxsackie virus (CV) A16, CV-A6 and CV-A10, as well as other relatively rare serotypes including CV-A4, CV-A2 and CV-A8. Of these, EV-A71 accounts for the largest proportion of cases with unfavorable clinical outcomes, but the frequency of severe disease caused by other serotypes has been increasing recently [3, 4]. There are frequent HFMD epidemics in the Asia Pacific region, posing a significant threat to public health [2, 3, 5].

The exact mechanism of severe HFMD pathogenesis remains to be fully elucidated. Previous studies suggest that enteroviruses invade and replicate in the central nervous system, initiating a damaging inflammatory response. Immunopathological injury also affects the cardiopulmonary system [5]. There are yet no effective antiviral drugs targeting HFMD associated enteroviruses, although significant efforts have been made to understand virus-human host interactions in order to identify promising candidates [6]. The role of viral load in the progress of HFMD and development of complications is of significance to both clinicians and researchers alike. In an animal model, faster EV-A71 replication was associated with severe disease [7], implying that pathology is primarily driven by the virus. However, the relationship between viral load and clinical disease severity has been rarely studied in HFMD cases to date. Real-time RT-PCR (reverse transcription-polymerase chain reaction) has been widely used in HFMD related studies, amplification cycle threshold (Ct) values of which have been used to indirectly measure viral load as an indicator of viral genomic load in other studies [8, 9]. Based on a prospective clinical study of HFMD cases at a children's hospital, we conducted this study to examine the associations of viral load with HFMD severity as well as other potential factors.

## 2 Methods

### 2.1 Study participants

The HFMD patients included in this analysis were recruited in a clinical study of HFMD at Henan Children's Hospital, which is a pediatric referral hospital in Zhengzhou of Henan province in central China. The clinical study has been introduced elsewhere [10]. In brief, all inpatient HFMD cases admitted to Henan Children's Hospital from February 15, 2017 to February 15, 2018 were invited to participate in the study. Demographics, past medical history, clinical data during hospitalization and clinical specimens on admission were prospectively collected. A total of 1840 HFMD cases were enrolled into the study, of whom only those with available RT-PCR Ct values for pan-enterovirus probes and primers from throat swabs collected on admission were included in this analysis. Data from patients where the enterovirus serotype could not be determined, where more than one serotype of enteroviruses was present, or the total number of cases infected with a given serotype was less than 20, were excluded from the analysis.

### 2.2 Outcome measures

The indicators used to define severe HFMD disease were as follows: CNS complications, including meningitis, encephalitis, brainstem encephalitis, encephalomyelitis, acute flaccid paralysis, autonomic nervous system (ANS) dysregulation and subsequent severe cardiopulmonary complications [11]; requirement for systemic corticosteroids or intravenous immunoglobulin (IVIG) during hospitalization, which are recommended for treatment of HFMD cases with encephalomyelitis and persistent high fever as well as critical HFMD cases [12]; admission to the intensive care unit (ICU) and length of hospital stay (LOS) more than 5 days.

### 2.3 Collection of throat swabs

Throat swabs were collected from enrolled HFMD cases within 48 h of admission. A standardized technique was used to collect the throat swabs. Throat swab collection was conducted by nurses, who were specially trained for this task by study staff beforehand. The lateral and posterior pharynx were swabbed two or three times without touching the tongue or buccal mucosa using a plastic shaft fiber swab with the aid of a tongue depressor and then the collected swab was immediately inserted into the conical tube with 3.5 mL of universal viral transport medium (UTM, Yocon, Beijing, China). All throat swabs were stored in the 4 °C refrigerator temporarily (no more than 24 h) before being preserved at −80 °C.

### 2.4 Virologic assay

RNA extractions and subsequent real-time RT-PCR were completed by two dedicated laboratory staff who were trained especially for this study at the Fudan University biosafety level 2 laboratory (Shanghai, China). Details of the RT-PCR assay have been described previously [10]. Briefly, RNA was extracted from specimens using QIAamp Viral RNA Mini Kit (QIAGEN, Hilden, Germany, Cat.#52906) and tested using real-time RT-PCR with primers for EV-A71, CV-A16 and pan-enterovirus (Quant one step qRT-PCR kit, Tiangen, China, Cat.#FP304–01), if EV-A71 or CV-A16 could not be identified, then further tested for specific enterovirus serotypes using several nested RT-PCRs. For nested RT-PCRs, the first-round amplification was performed in a 50 μL reaction volume using a SuperScript III One-Step RT-PCR Kit (Invitrogen, Carlsbad, CA, USA, Cat.#12574035), and the second round using DreamTaq Green PCR Master Mix (Invitrogen, Carlsbad, CA, USA, Cat.#K1082) in a 25 μL volume. The sequences obtained in this study were submitted to NCBI under accession numbers MW028180 - MW029376.

Each operation step, including preprocessing specimens, extracting RNA and amplifying RNA, was performed in different areas of the laboratory, to avoid false positive testing results. Enterovirus genomic load was measured using the number of amplification cycles, i.e. Ct value, required to produce a positive result in real-time RT-PCR. A Ct value of ≤38 under "S" melt curve was considered positive. In this analysis, Ct values of pan-enteroviruses probes and primers in real-time RT-PCR were used as a semiquantitative proxy for the inverse of relative viral genomic loads, as in other similar studies [8, 9].

### 2.5 Precision of Ct values and standard curves between relative viral genomic load and Ct values

Positive and negative controls were in place when extracting and amplifying RNA in our in-house laboratory system for the real-time RT-PCR. Across the whole testing period of the study, Ct values of the positive reagent control were highly reliable, mean ± SD (*n* = 34) of which were 9.09±0.77 for pan-enterovirus. The reliability of real-time RT-PCR lays the foundation for using Ct values as proxy of relative viral genomic load. In order to verify the association between Ct values and relative viral concentrations, we performed the real-time RT-PCR under the same conditions on serially diluted samples, which were collected from three patients and were 2-fold serially diluted with duplicate wells for each dilution. The Ct values for pan-enteroviruses probes and primers were shown to be positively linearly associated with the virus concentrations. Based on the standard curves produced by experiments, the relative genomic concentrations (C_rel,_ in arbitrary units) could be estimated with Ct values using the following formulas: log(C_rel_)=−0.311 × Ct (Supplementary Fig. 1).

### 2.6 Statistical analyses

The associations between two categorical variables were tested using chi-square tests and Fisher's exact tests. The associations between enterovirus genomic load in Ct values and binary variables were analysed using Wilcoxon rank-sum test. The associations between each serotype of enterovirus genomic load in Ct values and throat swab collection time after illness were analysed using the linear regression model. Each enterovirus serotype of HFMD cases were divided into three categories of viral genomic load (low, medium, high) according to Ct values which were classified into tertiles for each virus serotype, and associations between four binary clinical severity outcomes and viral genomic load categories were analysed using Cochran-Armitage Trend Test.

Logistic regression models were used to calculate the adjusted odds ratio (OR) of clinically more severe outcomes among viral genomic load categories (low, medium, high), controlling for age, residence type and parents’ highest education. These three factors were selected based on biological plausibility and their associations with both clinical severity and viral genomic load. Systemic corticosteroids treatment before throat swab collections, which might possibly increase the viral load of EV-A71 according to an animal study [13], was not included as a confounding factor in the logistic regression models for primary analysis, because our analyses demonstrated there was no association between systemic corticosteroids use before throat swab collection and viral load among clinically mild HFMD cases (Supplementary Table 1). In a sensitivity analysis, systemic corticosteroids use before throat swab collection and throat swab collection time respectively were added into the regression model to examine whether there was any major influence on the association between viral genomic load and clinical severity.

Given that it is possible that viral shedding in throat swabs might have stopped when viral shedding still continues in stool for some HFMD cases [14], who were actually laboratory confirmed HFMD cases but were excluded from this study for negative enterovirus testing results of throat swabs, the sensitivity analysis was performed by taking into account the virological testing results of stool at the hospital laboratory, where testing details were described previously and only two specific virus serotypes (EV-A71 and CV-A16) could be distinguished [10]. Those participants whose throat swabs tested negative for enteroviruses but whose stool tested positive for EV-A71 or CV-A16 were added into the analysis as HFMD cases infected with EV-A71 or CV-A16 and their Ct values were assigned 39, in order to examine whether there were any significant changes in the associations between enterovirus genomic load and throat swab collection time, clinical severity and other factors. All statistical tests were two-sided and p values ≤ 0.05 were considered statistically significant. R version 3.6.1 was used for all analyses.

### 2.7 Ethics

The study protocol was reviewed by the Institutional Review Boards of Henan Children's Hospital (IRB#YZ-17-006), Chinese center for Disease Prevention and Control (IRB#201624), and Public Health School of Fudan University (IRB#2017-12-0654). Written informed consent was provided by parents or legal guardians of study participants on enrollment.

### 2.8 Role of the funding sources

The funders of the study had no role in study design, data collection, data analysis, interpretation, or writing of the report.

## 3 Results

**Fig. 1:**
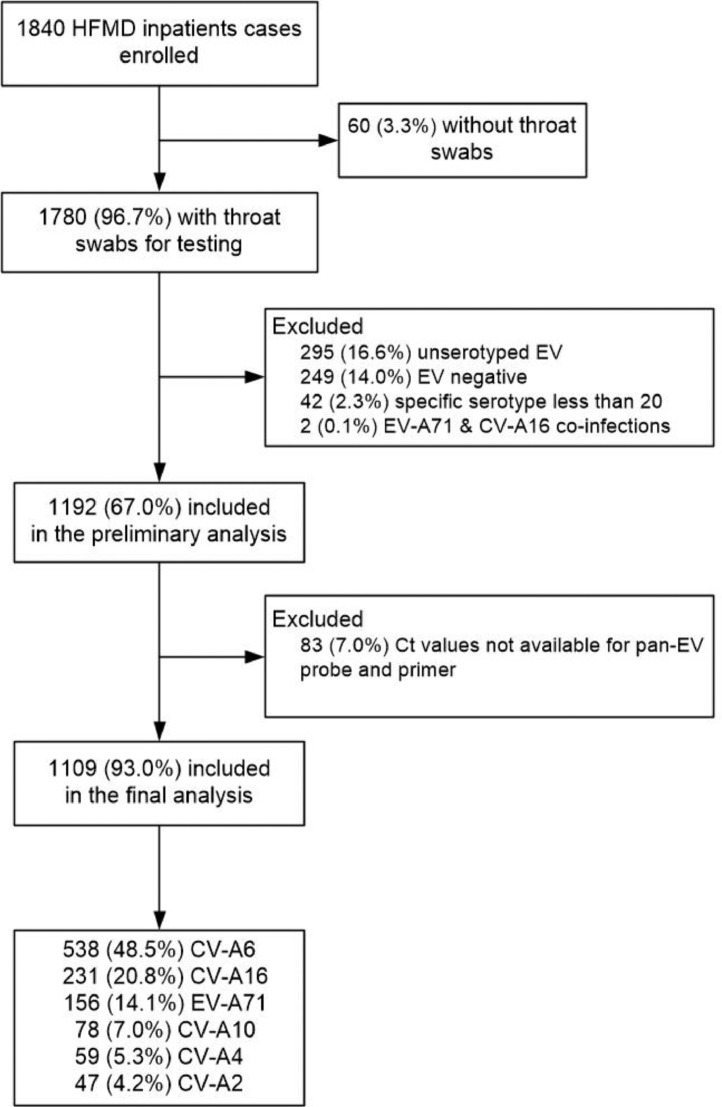
Flow diagram of including HFMD inpatient cases in the study.

During the study period, 1840 children were hospitalised with a clinical diagnosis of HFMD. Of these, 1109 had throat swabs available for diagnostic testing, tested positive for a specific enterovirus serotype, and were included in this study (Fig. 1). Less than 5% of included children had underlying medical conditions or low birth weight, while less than 10% of the study children were had premature births regardless of enterovirus serotypes, and no patient had an underlying medical condition causing immunocompromise (Table 1). Forty-two percent of patients testing positive for EV-A71 were less than 2 years of age; for the other serotypes more than half of patients were less than 2 years. The proportion of patients needing ICU admission was less than 5% for all serotypes apart from EV-A71, whereas the proportion of patients with CNS complications, requiring systemic corticosteroids or IVIG, or with length of stay > 5 days (the other three markers of severe disease) was generally larger than 5% for all serotypes. The participants included in the analysis were slightly older and more likely to reside in rural areas than those who were excluded from analysis. Other demographic characteristics, past medical history and clinical severity were similar (Supplementary Table 2).

**Table 1:** Characteristics of HFMD inpatient cases by enterovirus serotype included in the study.

Characteristics	EV-A71 (*N* = 156)	CV-A6 (*N* = 538)	CV-A16 (*N* = 231)	CV-A10 (*N* = 78)	CV-A4 (*N* = 59)	CV-A2 (*N* = 47)	P values
Age, years	2.2 (1.5–3.4)	1.4 (1.1- 2.3)	1.8 (1.4–2.7)	1.6 (1.2–2.3)	1.7 (1.3–2.7)	1.5 (1.2–2.3)	<0.001
Age group							
0–1 years	65 (42)	375 (70)	130 (56)	53 (68)	39 (66)	31 (66)	<0.001
2–14 years	91 (58)	163 (30)	101 (44)	25 (32)	20 (34)	16 (34)	
Male	100 (64)	332 (62)	156 (68)	50 (64)	40 (68)	34 (72)	0.51
Rural residence	59 (38)	140 (26)	81 (35)	30 (38)	18 (31)	5 (11)	<0.001
Parents’ education							
High School or below	76 (49)	190 (35)	93 (40)	31 (40)	25 (42)	11 (23)	0.013
Junior College or above	80 (51)	348 (65)	138 (60)	47 (60)	34 (58)	36 (77)	
Underlying medical conditions*	5 (3)	7 (1)	3 (1)	2 (3)	2 (3)	0 (0)	0.33
Premature birth†	7 (4)	18 (3)	14 (6)	6 (8)	2 (3)	4 (9)	0.25
Low birthweight‡	6 (4)	15 (3)	9 (4)	3 (4)	2 (3)	1 (2)	0.95
Major feeding ways within 6 months after birth							
Breast feeding	115 (74)	383 (71)	171 (74)	52 (67)	44 (75)	35 (74)	0.81
Non-breast feeding	41 (26)	155 (29)	60 (26)	26 (33)	15 (25)	12 (26)	
Diagnosed as CNS complications	63 (40)	38 (7)	8 (3)	8 (10)	10 (17)	6 (13)	<0.001
Require special treatment§	74 (47)	52 (10)	15 (6)	8 (10)	11 (19)	6 (13)	<0.001
ICU admission	32 (21)	7 (1)	2 (1)	3 (4)	2 (3)	0 (0)	<0.001
LOS>5 days	68 (44)	79 (15)	26 (11)	13 (17)	12 (20)	5 (11)	<0.001

Data were n (%) or median (IQR). Abbreviations: CNS, central nervous system; ICU, intensive care unit; LOS, length of stay. P-values were estimated by Fisher's exact test (ICU admission, underlying medical conditions, premature birth and low birthweight), chi-squared tests (all other categorical characteristics) and Wilcoxon rank-sum test (age).

⁎ Underlying medical conditions included epilepsy (4), brain damage (3), rickets (3), adenoids hypertrophy (3), delayed milestone (2), cranial nerve injury (1), progressive muscular dystrophy (1), tuberous sclerosis (1) and fused kidney (1).

† Defined as born before 37 weeks of gestation.

‡ Defined as birth weight which was below 2500 g.

§ Defined as receiving systemic corticosteroids or IVIG during hospitalization.

**Fig. 2:**
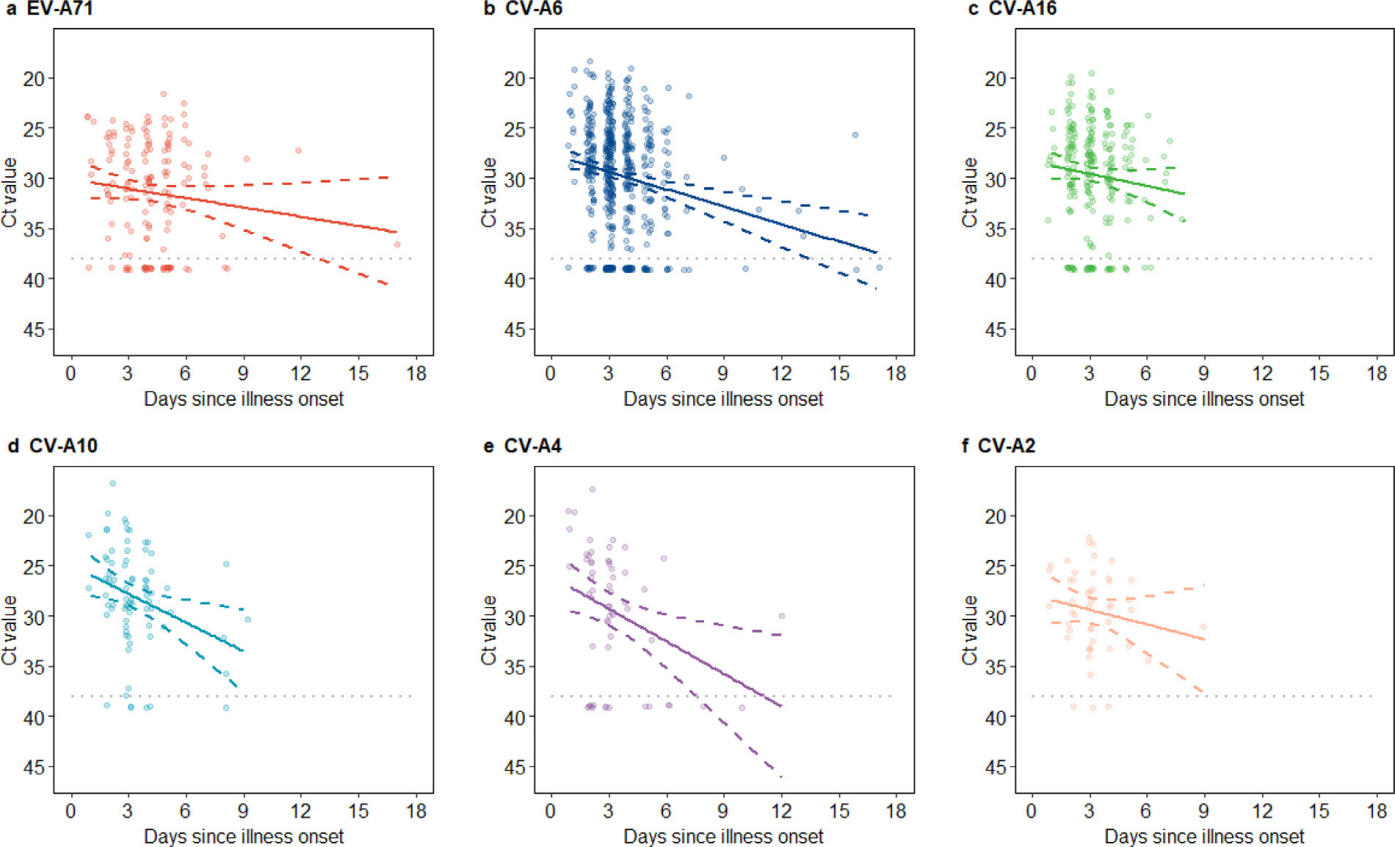
The association between virus genomic load and throat swab collection time since illness onset by enterovirus serotype. (a) EV-A71. (b) CV-A6. (c) CV-A16. (d) CV-A10. (e) CV-A4. (f) CV-A2. The line represented the linear regression line and the dashed line represented 95% confidence interval. Lower cycle threshold (Ct) values indicated higher virus genomic loads. The gray dotted line denoted the threshold of Ct value for determining positive in real time RT-PCR.

Viral load appeared to decline with increasing time between illness onset and throat swab collection for all six serotypes (Fig. 2). This association was statistically significant for CV-A6, CV-A10 and CV-A4, but was not significant for EV-A71, CV-A16 and CV-A2 in the primary analysis (Supplementary Table 3). However, in the sensitivity analysis accounting for those whose throat swabs were negative but stools tested positive for EV-A71 or CV-A16, viral genomic loads of both EV-A71 and CV-A16 still appeared to decline as time increased between illness onset and throat swab collection (Supplementary Fig. 2), while the slope of decline for these serotypes became statistically significant (Supplementary Table 3). Importantly, the timing of throat swab collection after illness onset was similar across different clinical severities within each serotype (Supplementary Table 4). Children who were normal birthweight, who resided in urban area and whose parents received higher education appeared to have slightly higher viral genomic loads across all six serotypes, but most of these differences were not statistically significant (Table 2). In the sensitivity analysis accounting for those whose throat swabs were negative but stools tested positive for EV-A71 or CV-A16, the results were similar to those of the primary analyses (Supplementary Table 5).

**Table 2:** Viral genomic load (Ct values) of throat swabs by characteristic by enterovirus serotype.

Characteristics	Ct value, median (IQR)					
	EV-A71 (*N* = 156)	CV-A6 (*N* = 538)	CV-A16 (*N* = 231)	CV-A10 (*N* = 78)	CV-A4 (*N* = 59)	CV-A2 (*N* = 47)
Age group						
0–1 years	31 (27–36)	29 (26–33)	29 (27–33)	29 (26–32)	28 (25–36)	29 (26–32)
2–14 years	30 (27–37)	29 (25–33)	28 (26–31)	26 (24–29)	30 (25–34)	29 (27–32)
P value	0.54	0.73	0.053	0.023	0.75	0.78
Sex						
Male	30 (27–35)	29 (26–33)	28 (26–32)	28 (25–30)	28 (25–39)	29 (26–33)
Female	31 (27–39)	29 (26–33)	29 (26–35)	28 (26–30)	29 (24–33)	29 (26–31)
P value	0.13	0.56	0.21	0.85	0.76	0.80
Residence type						
Rural	32 (29–39)	29 (26–33)	30 (27–35)	27 (25–29)	29 (25–39)	33 (29–34)
Urban	30 (26–35)	29 (26–33)	28 (26–31)	29 (25–31)	28 (25–32)	29 (26–31)
P value	0.023	0.76	0.025	0.21	0.19	0.30
Parents’ highest education						
High school or below	31 (28–37)	29 (26–33)	29 (26–34)	28 (25–32)	30 (26–33)	31 (28–34)
Junior college or above	30 (27–36)	29 (26–33)	28 (26–31)	28 (24–30)	28 (25–37)	29 (26–32)
P value	0.24	0.54	0.37	0.37	0.49	0.37
Underlying medical conditions						
No	31 (27–37)	29 (26–33)	29 (26–32)	28 (25–31)	29 (25–39)	29 (26–32)
Yes	30 (29–35)	31 (31–36)	32 (29–35)	26 (24–28)	26 (25–27)	…
P value	0.87	0.17	0.57	0.68	0.48	…
Premature birth*						
No	30 (27–37)	29 (26–33)	28 (26–32)	28 (25–31)	28 (25–33)	29 (26–32)
Yes	31 (26–32)	31 (27–38)	30 (28–34)	29 (27–29)	34 (32–37)	30 (27–33)
P value	0.38	0.064	0.13	0.61	0.31	0.88
Low birthweight†						
No	30 (27–37)	29 (26–33)	29 (26–32)	28 (25–30)	28 (25–33)	29 (26–32)
Yes	31 (28–34)	32 (29–39)	29 (28–30)	29 (26–34)	34 (32–37)	31 (31–31)
P value	0.78	0.024	0.62	0.66	0.31	0.61
Major feeding ways within 6 months after birth						
Non-breast feeding	30 (26–34)	28 (26–32)	29 (27–33)	29 (25–30)	28 (24–31)	31 (28–32)
Breastfeeding	31 (27–37)	29 (26–34)	28 (26–32)	27 (24–30)	28 (25–39)	29 (26–32)
P value	0.30	0.075	0.16	0.39	0.49	0.34

Data were median (IQR). P-values were estimated by Wilcoxon rank-sum test. Abbreviations: Ct, cycle threshold; IQR, interquartile range.

⁎ Defined as born before 37 weeks of gestation.

† Defined as birth weight which was below 2500 g.

**Fig. 3:**
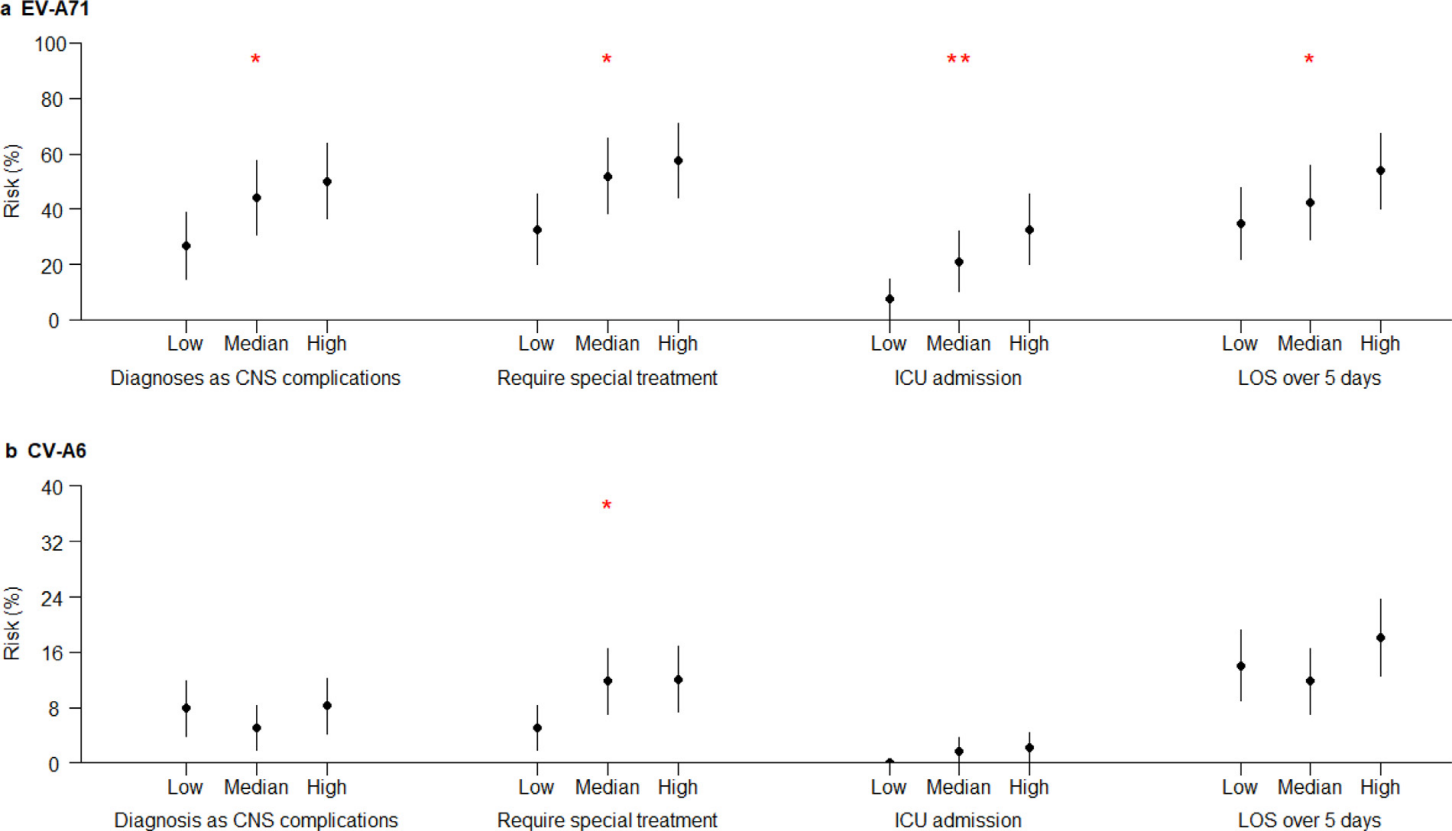
Genomic load categories and risks of four clinically more severe outcomes among HFMD cases by enterovirus serotype. (a) EV-A71. (b) CV-A6. The point indicated risks of clinically more severe outcomes and the line indicated the 95% confidence interval. The one and two red stars indicated p values less than 0.05 and 0.01, respectively, using Cochran-Armitage Trend Test. Genomic load categories of EV-A71 were classified based on Ct values as follows: low, ≥34.4; medium, 28.3–34.3; and high, <28.3. Genomic load categories of CV-A6 were classified based on Ct values as follows: low,≥31.2; medium, 26.7–31.1; and high, <26.7.

There was a trend for the risks of all four clinically severe outcomes to increase with the increase in EV-A71 genomic load categories (Fig. 3). The associations were statistically significant for risks of diagnoses of CNS complications (*p* = 0.016, Cochran-Armitage Trend Test), the need for systemic corticosteroids or IVIG (*p* = 0.011, Cochran-Armitage Trend Test), ICU admission (*p* = 0.002, Cochran-Armitage Trend Test) and LOS over 5 days (*p* = 0.048, Cochran-Armitage Trend Test). The similar statistically significant associations also existed for HFMD cases infected with CV-A6 between viral genomic load and the need for systemic corticosteroids or IVIG (*p* = 0.024, Cochran-Armitage Trend Test). No similar trends were found between the risks of clinically severe outcomes and viral genomic load categories among HFMD cases infected with CV-A16, CV-10, CV-A4 or CV-A2, and their associations were not statistically significant (Supplementary Fig. 3).

In the multivariate analyses adjusting for age, residence type and parents’ highest education, there was a trend for point estimates of adjusted ORs to increase with viral genomic load categories for all four markers of severe disease in children infected with EV-A71. The associations between relative high viral genomic load and risks of severe diseases were statistically significant in children with EV-A71 for all four markers of severe disease (Table 3). Similarly, an increased need for systemic corticosteroids or IVIG in those with higher viral genomic load remained after adjustment in patients infected with CV-A6; adjusted ORs for ICU admission could not be calculated because no cases in low genomic load category were admitted to ICU. There were no significant associations between the four markers of severe disease and viral genomic load for CV-A16, CV-A10, CV-A4 and CV-A2 (Supplementary Table 6).

**Table 3:** Adjusted associations of genomic load categories with risks of clinically more severe outcomes for EV-A71 and CV-A6.

Outcome Genomic load category*	EV-A71		CV-A6	
	Adjusted OR†	P value	Adjusted OR†	P value
Diagnoses as CNS complications				
Low	Reference	…	Reference	…
Medium	2.16 (0.93–4.99)	0.07	0.61 (0.25–1.46)	0.27
High	3.42 (1.44–8.12)	0.005	1.06 (0.49–2.28)	0.89
Require special treatment‡				
Low	Reference	…	Reference	…
Medium	2.23 (1.00–4.99)	0.05	2.50 (1.11–5.64)	0.03
High	3.27 (1.42–7.51)	0.005	2.59 (1.16–5.81)	0.02
ICU admission				
Low	Reference	…	Reference	…
Medium	3.26 (0.95–11.20)	0.06	NA§	NA§
High	7.73 (2.25–26.53)	0.001	NA§	NA§
LOS over 5 days				
Low	Reference	…	Reference	…
Medium	1.37 (0.61–3.07)	0.44	0.84 (0.45–1.56)	0.58
High	2.57 (1.13–5.84)	0.02	1.36 (0.77–2.40)	0.29

Abbreviations: Ct, cycle threshold; OR, odds ratio; CNS, central nervous system; ICU, intensive care unit; LOS, length of stay.

⁎ Genomic load categories of EV-A71 were classified based on Ct values as follows: low,≥34.4; medium, 28.3–34.3; and high, <28.3. Genomic load categories of CV-A6 were classified based on Ct values as follows: low,≥31.2; medium, 26.7–31.1; and high, <26.7.

† Odds ratio adjusted for age, residence type and parents’ highest education.

‡ Defined as receiving systemic corticosteroids or IVIG during hospitalization.

§ No case of low genomic load admitted to ICU, thus no reliable model could be established.

In the sensitivity analysis which included time interval between illness onset and specimen collection in the multivariate regression model, adjusted ORs between relative genomic load groups and risks of severe HFMD cases changed only slightly compared with the findings for EV-A71 and CV-A6 in the primary analysis (Supplementary Table 7). In the sensitivity analysis which included systemic corticosteroids use before specimen collection into the multivariate regression model, the association between high viral genomic load groups and increased risks of severe HFMD cases remained for EV-A71, similar to the findings of the primary analyses (Supplementary Table 8), but the association between viral genomic group and need for special treatment was no longer significant for CV-A6. In the sensitivity analyses for HFMD cases infected with EV-A71 and CV-A16, the increasing risks of clinically severe disease with higher viral genomic load remained for EV-A71, but were still not observed for CV-A16 (Supplementary Fig. 4. and Supplementary Table 9), similar to the findings of the primary analyses.

## 4 Discussion

In this hospital-based prospective study, we observed that four markers of disease severity (CNS complications, the need for systemic corticosteroids or IVIG, admission to ICU and LOS over 5 days) were more frequent in patients infected with EV-A71 with higher viral genomic load (as measured by RT-PCR cycle threshold). Similarly, the need for systemic corticosteroids or IVIG treatment also appeared to increase with the increase in viral genomic loads of CV-A6, but the association was not as consistent as for EV-A71 in a sensitivity analysis. We also observed that viral genomic loads of HFMD associated enteroviruses, including not only the predominant serotypes of CV-A6 and CV-A10, but also the relatively rare serotype of CV-A4, tended to decline over time after illness onset. This is the one of the few studies to examine associations between enterovirus genomic loads and clinical severities among HFMD cases.

The current evidence suggests that enteroviruses initially replicate in the oropharyngeal cavity and small bowel and then cause a mild viremia. Thereafter, enteroviruses disseminate to, and replicate in, other organs and mucous membranes, causing evident clinical illness [5]. A minority of HFMD cases may develop CNS complications; in the cases spread of the virus into the CNS is presumed to occur. The exact route of viral invasion into CNS is unclear but likely involves the movement of enteroviruses along cranial and peripheral nerves toward CNS [15, 16]. Tonsils are an important site outside the CNS for viral replication in HFMD [17], thus viral genomic load in throat swabs theoretically correlates with viral burden. In animal models, EV-A71 viral load is lower in surviving animals [18, 19]. Therefore, we hypothesised that increasing viral load measured in throat swabs would correlate with the clinical severity of HFMD. Our findings confirm this association between viral load and clinical severity for EV-A71. Given severe HFMD patients may have long term sequelae [20], it would be interesting to examine the association between viral load in the acute phase and risk of long term sequelae.

In this study we used four indicators to measure the clinical severity of HFMD, but these four indicators may differ in their relationship with disease severity. For instance, the LOS can be affected by factors other than illness severity [21, 22]. Nevertheless, HFMD cases with more severe illness generally tended to have a significantly longer LOS than those patients with milder illness, as observed in another multi-center study recruiting 466 HFMD patients in Vietnam [23]. The findings that viral genomic load was associated with all four indicators of clinical severity for EV-A71 increased our confidence in the validity of the association. However, these findings are inconsistent with several other Chinese studies which failed to detect statistically significant differences, although clinically severe cases often showed a trend towards higher viral load [24]. Our study is the largest to date, therefore previous findings may be explained by insufficient sample size, or a lack of adequate assessment of the impact of specimen collection time.

In our study, the association of viral genomic loads with disease severity was only significant for EV-A71, and was not consistently statistically significant for other enterovirus serotypes. This was likely due to insufficient sample size; the case numbers for CV-A10, CV-4 and CV-A2 were all less than 100. On the other hand, clinically severe disease was rare for CV-A6 and CV-A16, despite the large number of cases observed for this serotype. Alternatively, it is possible other non-EV-A71 enteroviruses might have exhibited a different disease mechanism; most past pathological studies focused on EV-A71 and have rarely studied other serotypes. Therefore, clinico-pathological studies of non-EV-A71 enteroviruses with larger sample sizes are warranted in the future, and are particularly important given that monovalent EV-A71 vaccine is available and effective in real world use [10].

Our study findings demonstrated viral load appeared to decline over time after illness onset for all the six serotypes; however, this association was statistically significant for CV-A6, CV-A10 and CV-A4, but was not significant for EV-A71, CV-A16 and CV-A2 in the primary analysis. Failure to detect a significant association between viral genomic load and throat swab collection time might be attributed to the exclusion of those whose viral shedding had stopped at the early phase of illness or relatively small sample size. Therefore, in the sensitivity analysis, we added the study subjects whose throat swabs tested negative for enteroviruses but whose stool tested positive for EV-A71 or CV-A16 back into the analysis, allowing us to detect a statistically significant difference. The comparison of point estimates of rate of decline between virus serotypes in the primary analysis suggested that viral load of EV-A71 and CV-A16 may decline more slowly than other serotypes CV-A6. This is consistent with the results of previous viral shedding studies, which indicated that shedding of EV-A71 and CV-A16 declined relatively more slowly than CV-A6 [25, 26], increasing confidence in our findings.

This is a comprehensive study on the association of viral load with HFMD severity as well as other potential factors, but several limitations need to be noted. Firstly, viral load was not measured using typical plaque assay or quantitative RT-PCR but quantified with relative viral genomic load based on Ct values as proxy instead. Nevertheless, our laboratory data confirmed the stability of our in-house assay system and linear associations between Ct values and RNA concentrations, which enhanced the robustness of quantifying relative viral genomic load based on Ct values. Moreover, in our study throat swabs were collected following a standardized quantitative protocol and collection operations were performed by well trained and qualified study nurses, which minimized the variability of viral genomic load attributed to specimen collections and thus lent us more confidence that viral genomic load measured in our laboratory reflected that in patients. Secondly, although our study showed viral genomic load appeared to decrease in throat swabs over time after illness onset, throat swabs were only collected at a single time point per patient, rather than at multiple time points. The dynamics of viral genomic loads need to be further explored in longitudinal studies. Thirdly, given our study subjects were hospitalised HFMD cases, and HFMD cases included in the analysis were slightly older and more likely to reside in rural areas than those who were excluded, our study findings may not necessarily apply to outpatient HFMD cases, community cases or other more general population. Nevertheless, the priority of HFMD management focuses on clinical severe cases, therefore our findings are of significant clinical relevance. Finally, our study was conducted at a single hospital. In future, similar studies should be conducted in multiple hospitals to verify the generalisability of our findings.

In conclusion, our study confirms that the severity of HFMD is correlated with viral genomic loads of EV-A71 in throat swabs. Such associations might exist for CV-A6, and need to be verified in another study with a larger number of CV-A6 cases. Viral genomic load in throat swabs might be negatively associated with time of specimen collection after illness onset for laboratory confirmed HFMD cases. Specific antiviral drugs should be developed to reduce enterovirus load, such measures would be expected to reduce the clinical severity of HFMD.

## Contributors

HJY, YL, and BJC designed the study. HJY and BJC supervised the study. CLS, JY, YL, YBC, CCZ, PC, LL, FW, CG, MYZ, and LL collected data and specimens. YHZ and QQ performed virologic testing. YHZ, QQ, YL, PC, CT, LL, CG, TCZ, KW, and MYZ cleaned data. YL, and LL analysed the data. YL, CLS, BJC, PW, and HJY wrote the drafts of the manuscript. CLS, HJY, YL, JY, BJC and MP interpreted the findings. MP, FW, LT and YBC commented on and revised drafts of the manuscript. All authors read and approved the final report.

## Declaration of Competing Interests

HJY has received research funding from Sanofi Pasteur, GlaxoSmithKline, Yichang HEC Changjiang Pharmaceutical Company and Shanghai Roche Pharmaceutical Company, outside the submitted work. BJC has received honoraria from Roche and Sanofi Pasteur, outside the submitted work. All other authors declare no competing interests.

## Appendix Supplementary materials

Image, application 1
