# Supplementary material for: Enterovirus genomic load and disease severity among children hospitalised with hand, foot and mouth disease
Source: eBioMedicine. 2020 Nov 6;62:103078. doi: 10.1016/j.ebiom.2020.103078 (PMC7653080; doi:10.1016/j.ebiom.2020.103078)
Supplement: Supplementary file 1 [file mmc1.docx]

**Supplementary material**


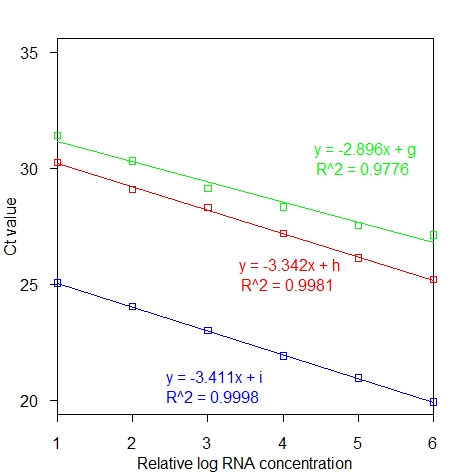


**Supplementary Fig. 1.** Standard curves between relative genomic concentrations and Ct values for pan-enterovirus primers and probes.


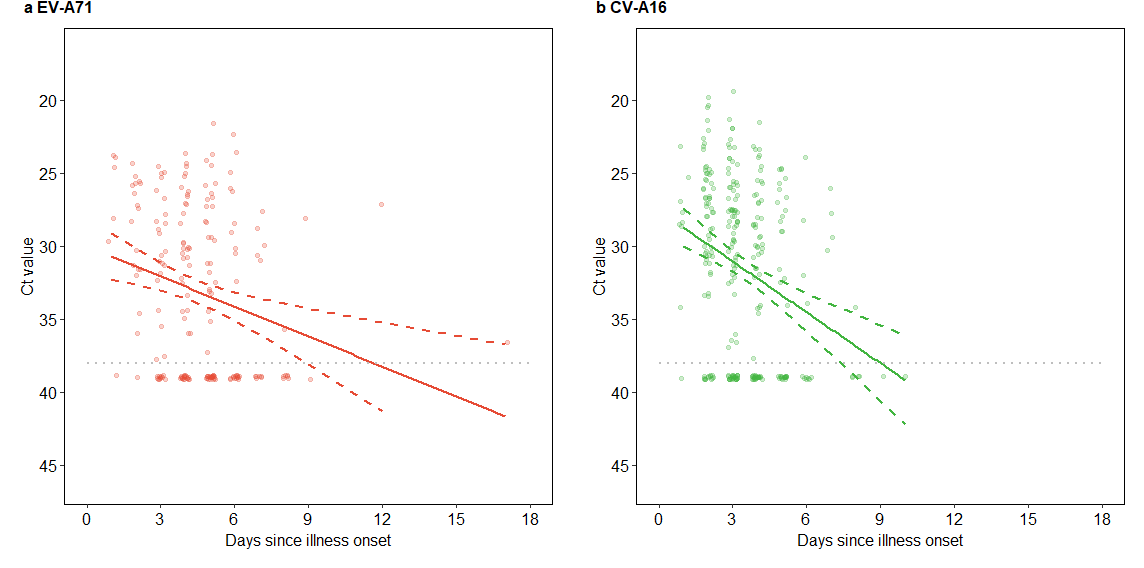


**Supplementary Fig. 2.** The association between virus genomic load and throat swab collection time since illness onset in the sensitivity analysis by enterovirus serotype. (a) EV-A71. (b) CV-A16.

The line represented the linear regression line and the dashed line represented 95% confidence interval. Lower cycle threshold (Ct) values indicated higher virus genomic loads. The gray dotted line denoted the threshold of Ct value for determining positive in real time RT-PCR.


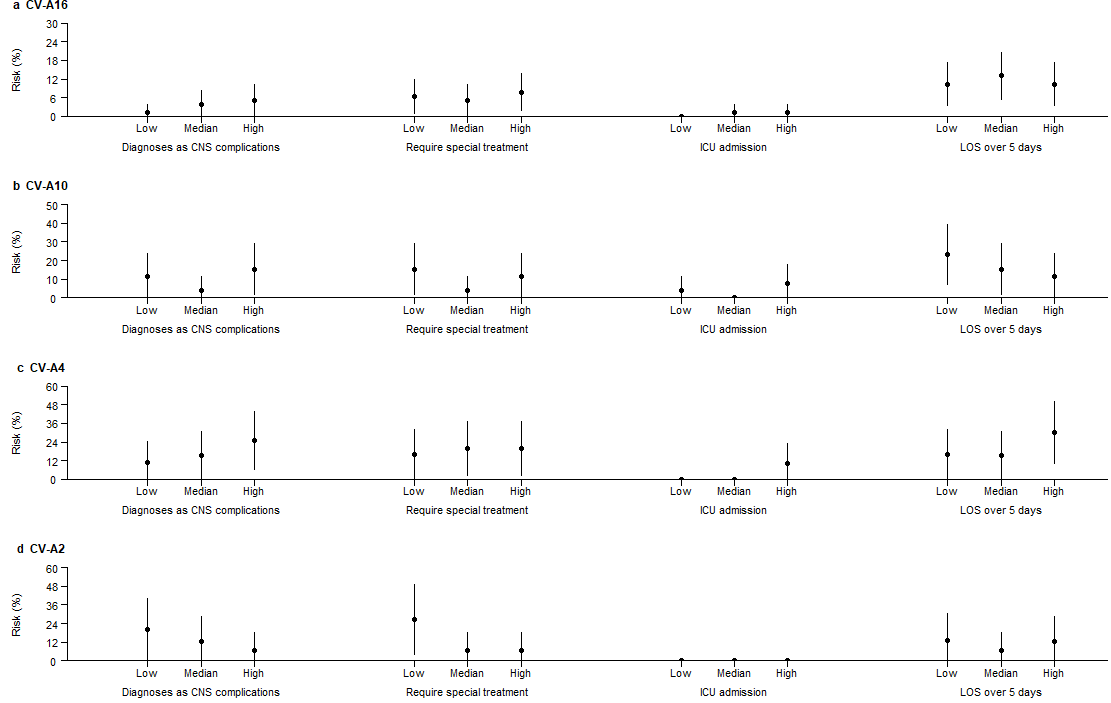


**Supplementary Fig. 3.** Genomic load categories and risk of four clinically more severe outcomes among HFMD cases infected with (A) CV-A16 (B) CV-A10 (C) CV-A4 (D) CV-A2.

The point indicated risks of clinically more severe outcomes and the line indicated the 95% confidence interval. Genomic load categories of CV-A16 were classified based on Ct values as follows: low, ≥30.7; intermediate, 26.9–30.6; and high, <26.9. Genomic load categories of CV-A10 were classified based on Ct values as follows: low, ≥29.3; intermediate, 26.3–29.2; and high, <26.3 Genomic load categories of CV-A4 were classified based on Ct values as follows: low, ≥32.2; intermediate, 25.5–32.1; and high, <25.5 Genomic load categories of CV-A2 were classified based on Ct values as follows: low, ≥31.3; intermediate, 27.7–31.2; and high, <27.7.


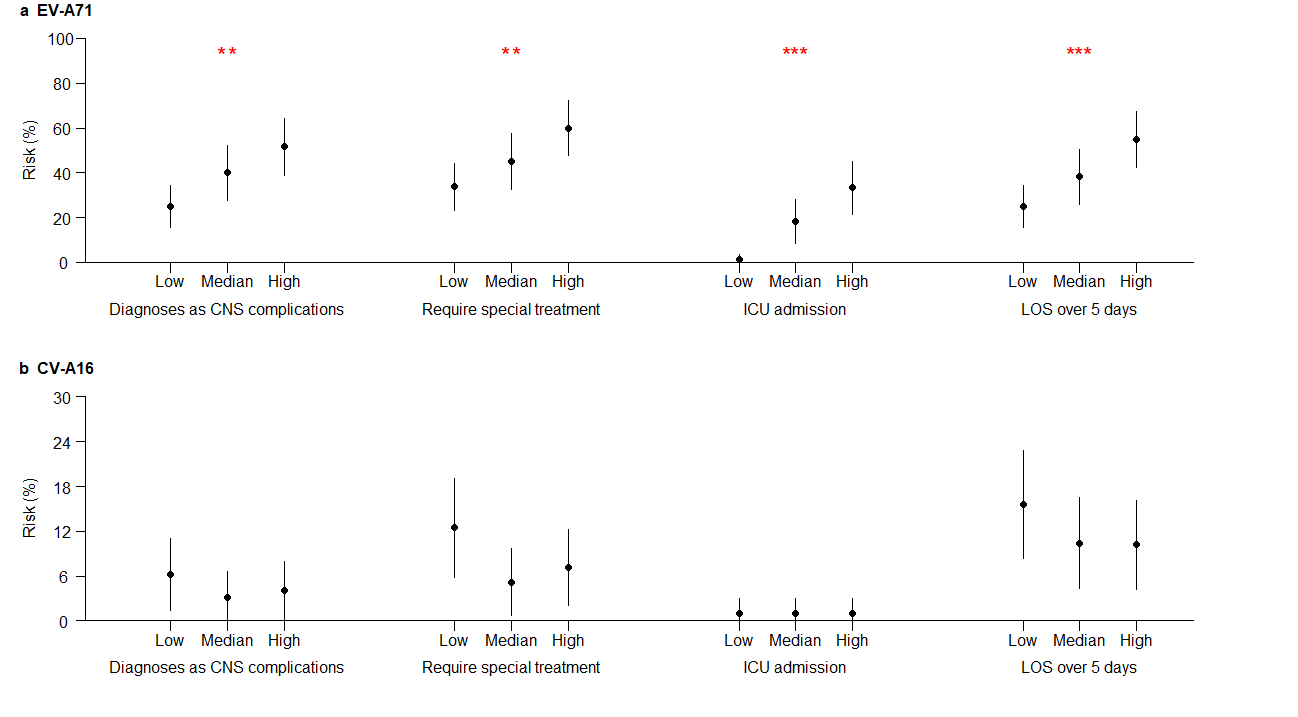


**Supplementary Fig. 4.** Genomic load categories and risks of four clinically more severe outcomes cases in the sensitivity analysis by enterovirus serotype. (a) EV-A71. (b) CV-A16.

The point indicated risks of clinically more severe outcomes and the line indicated the 95% confidence interval. The one, two and three red stars indicated p values less than 0.05, 0.01 and 0.001, respectively, using Cochran-Armitage Trend Test. Genomic load categories of EV-A71 were classified based on Ct values as follows: low, ≥39; intermediate, 29.0–38.9; and high, <29.0. Genomic load categories of CV-A16 were classified based on Ct values as follows: low, ≥37.8; intermediate, 27.8–37.7; and high, <27.8.

**Supplementary Table 1.**

Associations between viral genomic loads (Ct values) and corticosteroid use before specimen collection among mild laboratory HFMD cases.

| Definition of mild cases  Steroid use | Ct value, median (IQR) | | | | | | |
| --- | --- | --- | --- | --- | --- | --- | --- |
|  | EV-A71 | CV-A6 | CV-A16 | CV-A10 | CV-A4 | CV-A2 | Total |
| Cases with no CNS complications (N=976) |  |  |  |  |  |  |  |
| Steroid use | 38 (28, 39) | 29 (25, 31) | 29 (27, 31) | 31 (30, 32) | 29 (27, 34) | 31 (31, 31) | 29 (26, 32) |
| No steroid use | 31 (28, 39) | 29 (26, 33) | 29 (26, 32) | 28 (25, 30) | 28 (25, 39) | 29 (26, 32) | 29 (26, 33) |
| P value | 0.22 | 0.15 | 0.98 | 0.10 | 0.54 | 0.36 | 0.66 |
| Cases not admitted to ICU (N=1063) |  |  |  |  |  |  |  |
| Steroid use | 32 (28, 39) | 28 (25, 31) | 28 (26, 31) | 30 (28, 32) | 29 (26, 34) | 32 (31, 32) | 29 (26, 32) |
| No steroid use | 31 (28, 39) | 29 (26, 33) | 29 (26, 32) | 28 (25, 30) | 28 (25, 39) | 29 (26, 31) | 29 (26, 33) |
| P value | 0.59 | 0.074 | 0.75 | 0.23 | 0.69 | 0.22 | 0.58 |
| Cases of LOS within 5 days (N=906) |  |  |  |  |  |  |  |
| Steroid use | 33 (29, 39) | 29 (25, 31) | 27 (26, 31) | 30 (27, 32) | 29 (28, 31) | 31 (29, 32) | 29 (26, 32) |
| No steroid use | 31 (28, 39) | 29 (26, 33) | 29 (26, 32) | 28 (25, 30) | 28 (25, 39) | 29 (26, 33) | 29 (26, 33) |
| P value | 0.53 | 0.15 | 0.58 | 0.48 | 0.52 | 0.75 | 0.91 |

Abbreviations: Ct value, cycle threshold value; IQR, interquartile range; CNS, central nervous system; ICU, intensive care unit; LOS, lengths of stay. P values were calculated using Wilcoxon rank-sum test.

**Supplementary Table 2.**

Demographic characteristics and medical history of included and excluded participants from enrolled HFMD cases in a children’s hospital, February 2017-Februray 2018.

| Characteristics | Included (N=1109) | Excluded (N=731) | P value |
| --- | --- | --- | --- |
| Age, years | 1.6 (1.2-2.7) | 1.7 (1.2-2.9) | 0.296 |
| Male | 712 (64) | 450 (62) | 0.271 |
| Rural residence | 333 (30) | 255 (35) | 0.033 |
| Parents’ education |  |  | 0.189 |
| High School or below | 426 (38) | 304 (42) |  |
| Junior College or above | 683 (62) | 427 (58) |  |
| Underlying medical conditions^*^ | 19 (2) | 9 (1) | 0.527 |
| Premature birth^†^ | 51 (5) | 40 (5) | 0.462 |
| Low birthweight^‡^ | 36 (3) | 19 (3) | 0.511 |
| Diagnosed as CNS complications | 133 (12) | 105 (14) | 0.158 |
| Require special treatment^§^ | 166 (15) | 153 (21) | 0.001 |
| ICU admission | 46 (4) | 27 (4) | 0.714 |
| LOS>5 days | 203 (18) | 129 (18) | 0.766 |

Data were n (%) or median (IQR). P-values were estimated by Fisher’s exact test (ICU admission, underlying medical conditions, premature birth and low birthweight), chi-squared tests (all other categorical characteristics) and Wilcoxon rank-sum test (age). ^*^ Underlying medical conditions included epilepsy (5), rickets (3), brain damage (3), adenoids hypertrophy (3), delayed milestone (2), cranial nerve injury (1), progressive muscular dystrophy (1), anaemia (1), aniridia (1), fused kidney (1), tuberous sclerosis (1). ^†^ Defined as born before 37 weeks of gestation. ^‡^ Defined as birth weight which was below 2500g. ^§^ Defined as receiving systemic corticosteroids or IVIG during hospitalization.

**Supplementary Table 3.**

Estimates of slopes of the lines between Ct values and throat swab collection time by enterovirus serotype.

| Serotype | Change in Ct values (95% CI) per day since illness onset | | | | |
| --- | --- | --- | --- | --- | --- |
|  | Primary analysis | |  | Sensitivity analysis | |
|  | Coefficient | P value |  | Coefficient | P value |
| EV-A71 | 0.31 (-0.12,0.74) | 0.156 |  | 0.69 (0.29,1.08) | 0.001 |
| CV-A6 | 0.58 (0.31,0.85) | <0.001 |  | ... | ... |
| CV-A16 | 0.40 (-0.13,0.93) | 0.139 |  | 1.16 (0.71,1.61) | <0.001 |
| CV-A10 | 0.95 (0.25,1.65) | 0.01 |  | ... | ... |
| CV-A4 | 1.08 (0.31,1.86) | 0.008 |  | ... | ... |
| CV-A2 | 0.49 (-0.38,1.35) | 0.275 |  | ... | ... |

**Supplementary Table 4.**

Throat swab collection time by outcomes of clinical severity within each serotype.

| Outcomes of clinical severity | Throat swab collection time (days) after illness, median (IQR) | | | | | |
| --- | --- | --- | --- | --- | --- | --- |
|  | EV-A71  (N=156) | CV-A6  (N=538) | CV-A16  (N=231) | CV-A10  (N=78) | CV-A4  (N=59) | CV-A2  (N=47) |
| Diagnoses as CNS complications |  |  |  |  |  |  |
| No | 4 (3-5) | 3 (3-4) | 3 (2-4) | 3 (2-4) | 3 (2-3) | 3 (2-4) |
| Yes | 4 (3-5) | 3 (2-5) | 3 (2-4) | 3 (2-3) | 3 (1-6) | 3 (2-4) |
| P value | 0.26 | 0.91 | 1.0 | 0.11 | 0.74 | 0.86 |
| Require special treatment^*^ |  |  |  |  |  |  |
| No | 4 (3-5) | 3 (3-4) | 3 (2-4) | 3 (3-4) | 3 (2-3) | 3 (2-4) |
| Yes | 4 (3-5) | 3 (3-4) | 3 (2-4) | 2 (2-3) | 3 (2-3) | 2 (2-4) |
| P value | 0.94 | 0.61 | 0.52 | 0.077 | 0.87 | 0.75 |
| ICU admission |  |  |  |  |  |  |
| No | 4 (3-5) | 3 (3-4) | 3 (2-4) | 3 (2-4) | 3 (2-3) | 3 (2-4) |
| Yes | 4 (3-5) | 3 (2-4) | 4 (3-4) | 3 (2-6) | 1 (1-1) | ... |
| P value | 0.12 | 0.57 | 0.52 | 0.96 | 0.016 | ... |
| LOS over 5 days |  |  |  |  |  |  |
| No | 4 (3-5) | 3 (3-4) | 3 (2-4) | 3 (2-4) | 3 (2-3) | 3 (2-4) |
| Yes | 4 (3-5) | 3 (3-4) | 4 (2-4) | 3 (2-5) | 2 (2-5) | 3 (3-4) |
| P value | 0.21 | 0.35 | 0.073 | 0.74 | 0.95 | 0.46 |

Data were median (IQR). P-values were estimated by Wilcoxon rank-sum test. Abbreviations: CNS, central nervous system; ICU, intensive care unit; LOS, length of stay. ^*^ Defined as receiving systemic corticosteroids or IVIG during hospitalization.

**Supplementary Table 5.**

Viral genomic load (Ct values) of throat swabs by characteristic by enterovirus serotype.

| Characteristics | Ct value, median (IQR) | |
| --- | --- | --- |
|  | EV-A71 (N=200) | CV-A16 (N=290) |
| Age group |  |  |
| 0-1 years | 33 (28-39) | 31 (27-39) |
| 2-14 years | 33 (28-39) | 30 (26-39) |
| P value | 0.66 | 0.15 |
| Sex |  |  |
| Male | 32 (28-39) | 30 (27-39) |
| Female | 36 (28-39) | 31 (27-39) |
| P value | 0.17 | 0.11 |
| Residence type |  |  |
| Rural | 36 (30-39) | 33 (28-39) |
| Urban | 31 (27-39) | 30 (26-39) |
| P value | 0.044 | 0.041 |
| Parents’ education |  |  |
| High School or below | 34 (30-39) | 31 (27-39) |
| Junior College or above | 32 (27-39) | 30 (27-39) |
| P value | 0.35 | 0.46 |
| Underlying medical conditions |  |  |
| No | 32 (28-39) | 31 (27-39) |
| Yes | 35 (28-39) | 30 (27-39) |
| P value | 0.55 | 0.56 |
| Premature birth^*^ |  |  |
| No | 33 (28-39) | 30 (27-39) |
| Yes | 30 (29-35) | 32 (29-35) |
| P value | 0.40 | 0.87 |
| Low birth weight^†^ |  |  |
| No | 33 (28-39) | 30 (27-39) |
| Yes | 36 (30-39) | 30 (29-38) |
| P value | 0.73 | 0.51 |
| Major feeding ways within 6 months after birth |  |  |
| Non-breastfeeding | 33 (28-39) | 30 (27-39) |
| Breastfeeding | 33 (30-39) | 30 (28-39) |
| P value | 0.90 | 0.58 |

Data were median (IQR). P-values were estimated by Wilcoxon rank-sum test. Abbreviations: CNS, central nervous system; ICU, intensive care unit; LOS, length of stay. ^*^ Defined born before 37 weeks of gestation. ^†^ Defined as birth weight which is below 2500 g.

**Supplementary Table 6.**

Adjusted associations of genomic load categories with risks of clinically more severe outcomes for CV-A16, CV-A10, CV-A4 and CV-A2.

| Outcome  Genomic load category^*^ | CV-A16 | |  | CV-A10 | |  | CV-A4 | |  | CV-A2 | |
| --- | --- | --- | --- | --- | --- | --- | --- | --- | --- | --- | --- |
|  | Adjusted OR^†^ | P value |  | Adjusted OR^†^ | P value |  | Adjusted OR^†^ | P value |  | Adjusted OR^†^ | P value |
| Diagnoses as CNS complications |  |  |  |  |  |  |  |  |  |  |  |
| Low | Reference | ... |  | Reference | ... |  | Reference | ... |  | Reference | ... |
| Intermediate | 3.24 (0.32-32.94) | 0.32 |  | 0.17 (0.01-3.64) | 0.26 |  | 1.30 (0.17-9.75) | 0.80 |  | 0.67 (0.08-5.40) | 0.71 |
| High | 4.70 (0.50-44.67) | 0.18 |  | 1.42 (0.21-9.80) | 0.72 |  | 4.15 (0.60-28.76) | 0.15 |  | 0.34 (0.03-4.14) | 0.40 |
| Require special treatment^‡^ |  |  |  |  |  |  |  |  |  |  |  |
| Low | Reference | ... |  | Reference | ... |  | Reference | ... |  | Reference | ... |
| Intermediate | 0.96 (0.24-3.85) | 0.95 |  | 0.17 (0.01-2.23) | 0.18 |  | 1.28 (0.23-7.01) | 0.77 |  | 0.19 (0.02-2.03) | 0.17 |
| High | 1.63 (0.45-5.89) | 0.46 |  | 0.69 (0.11-4.27) | 0.69 |  | 1.32 (0.25-7.11) | 0.74 |  | 0.20 (0.02-2.16) | 0.19 |
| ICU admission |  |  |  |  |  |  |  |  |  |  |  |
| Low | Reference | ... |  | Reference | ... |  | Reference | ... |  | Reference | ... |
| Intermediate | NA^§^ | NA^§^ |  | NA^§^ | NA^§^ |  | NA^§^ | NA^§^ |  | NA^§^ | NA^§^ |
| High | NA^§^ | NA^§^ |  | 2.45 (0.14-42.83) | 0.54 |  | NA^§^ | NA^§^ |  | NA^§^ | NA^§^ |
| LOS over 5 days |  |  |  |  |  |  |  |  |  |  |  |
| Low | Reference | ... |  | Reference | ... |  | Reference | ... |  | Reference | ... |
| Intermediate | 1.38 (0.50-3.78) | 0.53 |  | 0.63 (0.14-2.88) | 0.55 |  | 0.91 (0.15-5.59) | 0.92 |  | 0.45 (0.03-6.02) | 0.55 |
| High | 1.08 (0.38-3.11) | 0.88 |  | 0.39 (0.08-2.04) | 0.26 |  | 2.88 (0.56-14.94) | 0.21 |  | 1.02 (0.11-9.18) | 0.99 |

Abbreviations: Ct, cycle threshold; OR, odds ratio; CNS, central nervous system; ICU, intensive care unit; LOS, length of stay.

^*^ Genomic load categories of CV-A16 were classified based on Ct values as follows: low, ≥30.7; intermediate, 26.9–30.6; and high, <26.9. Genomic load categories of CV-A10 were classified based on Ct values as follows: low, ≥29.3; intermediate, 26.3–29.2; and high, <26.3 Genomic load categories of CV-A4 were classified based on Ct values as follows: low, ≥32.2; intermediate, 25.5–32.1; and high, <25.5 Genomic load categories of CV-A2 were classified based on Ct values as follows: low, ≥31.3; intermediate, 27.7–31.2; and high, <27.7. ^†^ Odds ratio adjusted for age, residence type and parents’ highest education.

^‡^ Defined as receiving systemic corticosteroids or IVIG during hospitalization.

^§^ No case in the category admitted to ICU, thus no reliable model could be established.

**Supplementary Table 7.**

Adjusted associations of genomic load categories with risks of clinically more severe outcomes for EV-A71 and CV-A6 in the sensitivity analysis accounting for time interval between illness onset and specimen collection.

| Outcome  Genomic load category^*^ | EV-A71 | |  | CV-A6 | |
| --- | --- | --- | --- | --- | --- |
|  | Adjusted OR^†^ | P value |  | Adjusted OR^†^ | P value |
| Diagnoses as CNS complications |  |  |  |  |  |
| Low | Reference | ... |  | Reference | ... |
| Intermediate | 2.11 (0.91-4.90) | 0.08 |  | 0.62 (0.26-1.48) | 0.28 |
| High | 3.29 (1.38-7.86) | 0.0073 |  | 1.08 (0.50-2.35) | 0.85 |
| Require special treatment^‡^ |  |  |  |  |  |
| Low | Reference | ... |  | Reference | ... |
| Intermediate | 2.27 (1.01-5.11) | 0.046 |  | 2.55 (1.13-5.75) | 0.025 |
| High | 3.38 (1.46-7.87) | 0.0046 |  | 2.66 (1.18-5.99) | 0.018 |
| ICU admission |  |  |  |  |  |
| Low | Reference | ... |  | Reference | ... |
| Intermediate | 3.10 (0.90-10.73) | 0.073 |  | NA | NA |
| High | 7.24 (2.10-24.95) | 0.0017 |  | NA | NA |
| LOS over 5 days |  |  |  |  |  |
| Low | Reference | ... |  | Reference | ... |
| Intermediate | 1.32 (0.59-2.98) | 0.50 |  | 0.86 (0.46-1.60) | 0.62 |
| High | 2.43 (1.06-5.55) | 0.036 |  | 1.41 (0.80-2.51) | 0.24 |

Abbreviations: Ct, cycle threshold; OR, odds ratio; CNS, central nervous system; ICU, intensive care unit; LOS, length of stay.

^*^ Genomic load categories of EV-A71 were classified based on Ct values as follows: low,≥34.4; intermediate, 28.3–34.3; and high, <28.3. Genomic load categories of CV-A6 were classified based on Ct values as follows: low,≥31.2; intermediate, 26.7–31.1; and high, <26.7.

^†^ Odds ratio adjusted for age, residence type, parents’ highest education and time interval between illness onset and specimen collection.

^‡^ Defined as receiving systemic corticosteroids or IVIG during hospitalization.

^§^ No case of low genomic load admitted to ICU, thus no reliable model could be established.

**Supplementary Table 8.**

Adjusted associations of genomic load categories with risks of clinically more severe outcomes for EV-A71 and CV-A6 in the sensitivity analysis accounting for corticosteroid use before specimen collection.

| Outcome  Genomic load category^*^ | EV-A71 | |  | CV-A6 | |
| --- | --- | --- | --- | --- | --- |
|  | Adjusted OR^†^ | P value |  | Adjusted OR^†^ | P value |
| Diagnoses as CNS complications |  |  |  |  |  |
| Low | Reference | ... |  | Reference | ... |
| Intermediate | 2.55 (0.87-7.52) | 0.089 |  | 0.33 (0.12-0.91) | 0.033 |
| High | 4.23 (1.37-13.10) | 0.012 |  | 0.57 (0.23-1.41) | 0.22 |
| Require special treatment^‡^ |  |  |  |  |  |
| Low | Reference | ... |  | Reference | ... |
| Intermediate | 4.58 (1.07-19.58) | 0.040 |  | 1.43 (0.40-5.12) | 0.58 |
| High | 7.26 (1.62-32.61) | 0.0097 |  | 1.22 (0.34-4.43) | 0.76 |
| ICU admission |  |  |  |  |  |
| Low | Reference | ... |  | Reference | ... |
| Intermediate | 3.37 (0.90-12.65) | 0.072 |  | NA | NA |
| High | 8.12 (2.08-31.68) | 0.0026 |  | NA | NA |
| LOS over 5 days |  |  |  |  |  |
| Low | Reference | ... |  | Reference | ... |
| Intermediate | 1.23 (0.47-3.18) | 0.68 |  | 0.73 (0.38-1.39) | 0.34 |
| High | 2.44 (0.92-6.45) | 0.073 |  | 1.19 (0.67-2.14) | 0.55 |

Abbreviations: Ct, cycle threshold; OR, odds ratio; CNS, central nervous system; ICU, intensive care unit; LOS, length of stay.

^*^ Genomic load categories of EV-A71 were classified based on Ct values as follows: low,≥34.4; intermediate, 28.3–34.3; and high, <28.3. Genomic load categories of CV-A6 were classified based on Ct values as follows: low,≥31.2; intermediate, 26.7–31.1; and high, <26.7.

^†^ Odds ratio adjusted for age, residence type, parents’ highest education and corticosteroid use before specimen collection.

^‡^ Defined as receiving systemic corticosteroids or IVIG during hospitalization.

^§^ No case of low genomic load admitted to ICU, thus no reliable model could be established.

**Supplementary Table 9.**

Adjusted associations of genomic load categories with risks of clinically more severe outcomes for EV-A71 and CV-A16 in the sensitivity analysis.

| Outcome  Genomic load category^*^ | EV-A71 | |  | CV-A16 | |
| --- | --- | --- | --- | --- | --- |
|  | Adjusted OR^†^ | P value |  | Adjusted OR^†^ | P value |
| Diagnoses as CNS complications |  |  |  |  |  |
| Low | Reference | ... |  | Reference | ... |
| Intermediate | 1.93 (0.92-4.06) | 0.083 |  | 0.46 (0.11-1.92) | 0.29 |
| High | 4.03 (1.89-8.62) | <0.001 |  | 0.62 (0.17-2.30) | 0.48 |
| Require special treatment^‡^ |  |  |  |  |  |
| Low | Reference | ... |  | Reference | ... |
| Intermediate | 1.59 (0.79-3.22) | 0.20 |  | 0.39 (0.13-1.18) | 0.095 |
| High | 3.54 (1.71-7.31) | 0.001 |  | 0.59 (0.22-1.61) | 0.31 |
| ICU admission |  |  |  |  |  |
| Low | Reference | ... |  | Reference | ... |
| Intermediate | 17.27 (2.15-139.10) | 0.007 |  | 0.91 (0.06-14.95) | 0.95 |
| High | 50.22 (6.33-398.57) | <0.001 |  | 1.01 (0.06-17.21) | 0.99 |
| LOS over 5 days |  |  |  |  |  |
| Low | Reference | ... |  | Reference | ... |
| Intermediate | 1.89 (0.90-3.97) | 0.093 |  | 0.61 (0.26-1.45) | 0.26 |
| High | 4.43 (2.09-9.43) | <0.001 |  | 0.65 (0.27-1.55) | 0.33 |

Abbreviations: Ct, cycle threshold; OR, odds ratio; CNS, central nervous system; ICU, intensive care unit; LOS, length of stay. ^*^ Genomic load categories of EV-A71 were classified based on Ct values as follows: low, ≥39; intermediate, 29.0–38.9; and high, <29.0. Genomic load categories of CV-A16 were classified based on Ct values as follows: low, ≥37.8; intermediate, 27.8–37.7; and high, <27.8. ^†^ Odds ratio adjusted for age, residence type and parents’ highest education. ^‡^ Defined as receiving systemic corticosteroids or IVIG during hospitalization. ^§^ No case of low genomic load admitted to ICU, thus no reliable model could be established.
